# Development of Nanomaterials for Energy and Environmental Applications

**DOI:** 10.3390/molecules30081752

**Published:** 2025-04-14

**Authors:** Lin Ju

**Affiliations:** School of Physics and Electric Engineering, Anyang Normal University, Anyang 455000, China; julin@aynu.edu.cn

Globally, although rapid industry and economic growth have brought about remarkable social progress, they are also accompanied by serious environmental problems, such as the accumulation of fly ash, increased carbon dioxide emissions, and nitrogen pollution [1,2]. The solution to these problems urgently requires innovative clean energy technology. For example, the treatment and reuse of fly ash can reduce environmental pollution, while the reduction technology of carbon dioxide and nitrogen can help mitigate the greenhouse effect and the eutrophication of water bodies. In addition, hydrolysis technology can produce hydrogen as a clean fuel [3,4], and the development of supercapacitors and fuel cells provides new ways for the efficient storage and conversion of energy [5,6]. The progress of these technologies not only effectively responds to environmental degradation, but also promotes the transformation of energy structures and provides impetus for global sustainable development [7].

In this Special Issue, we focus on the development of nanomaterials for energy and environmental applications. We have selected ten excellent academic papers. Three of them provide an in-depth investigation of the method of carbon removal, four of them explore the process of hydrolysis, one article examines fuel cells in depth, another focuses on the study of N_2_ reduction, and the last article focuses on supercapacitors. We sincerely hope that this Special Issue can promote the development of nanomaterials in the field of environmental applications and energy sources, accelerate the innovation of new energy technology, and help the process of environmental pollution control.

**Water Splitting.** As a key technology for achieving a green hydrogen economy, the efficiency and economic viability of water splitting have been constrained by the high cost and performance limitations of traditional noble metal catalysts [8,9,10,11]. Four studies innovatively addressed these challenges through material design and synthesis strategies from different perspectives. Zhao et al. (Contribution 1) systematically introduced strategies, such as metal doping [12], metal nanoparticle incorporation [13], non-metal doping [14], and anion doping, which introduced discrete energy levels, enhanced visible light absorption, and promoted the separation and transport of photogenerated carriers [15]. This work provided theoretical foundations and experimental guidance for developing efficient photocatalysts for water-splitting reactions. Sun et al. (Contribution 2) constructed a low-cost Fe_2_O_3_-NiFe_2_O_4_ heterojunction composite using waste carbon fiber substrates [16]. The designed Fe_2_O_3_-NiFe_2_O_4_ nanocomposite demonstrates excellent hydrogen evolution reaction (HER) performance in 1 M KOH alkaline solution, with a low overpotential, a small Tafel slope, and robust stability. The synergistic effect between Fe_2_O_3_ nanorods and NiFe_2_O_4_ nanoparticles at the heterointerface significantly boosted HER activity. Cotton fiber cloth served as an effective substrate, facilitating composite growth and providing conductive pathways to enhance the HER process. This study offered a novel pathway for developing low-cost, efficient, and accessible HER electrocatalysts. Ummul et al. (Contribution 3) presented a method for synthesizing GaN:ZnO solid-solution materials, which used the visible-light-absorbing photocatalysts that are widely used in hydrogen production [17]. Leveraging the high reactivity of LiNH_2_ in molten LiCl, the method rapidly formed highly crystalline GaN:ZnO at 650 °C in just 2 h, outperforming traditional toxic gas-involving processes that require over 10 h. XRD, TEM, and XRF analyses confirmed the material formation under varied zinc sources and Zn/Ga ratios, with light absorption edges being tunable between 500 and 650 nm. This approach provided an efficient, safe, and environmentally friendly synthesis strategy for visible-light-driven applications. Lu et al. (Contribution 4) reported the hydrothermal synthesis of three-dimensional Ni-Mo-based nanoarrays that are uniformly grown on nickel foam [18]. The catalyst exhibited an outstanding oxygen evolution reaction (OER) activity and durability, achieving an overpotential of 296 mV and a low Tafel slope of 121 mV dec⁻^1^. Its large double-layer capacitance and abundant ion transport channels enhanced active sites and charge transfer efficiency, offering valuable insights for developing non-precious metal catalysts. Collectively, these studies advanced the development of efficient, stable, and economical water-splitting catalysts through structural engineering, interface optimization, and eco-friendly synthesis pathways. They provided multidimensional solutions for scaling up clean energy applications, driving progress toward a sustainable hydrogen economy.

**Carbon Removal.** With the acceleration of industrialization, the large-scale utilization of fossil energy around the world has caused an increase in environmental challenges. On one hand, the rapidly rising energy demand has led to a continuous surge in carbon dioxide emissions, exacerbating the greenhouse effect and the melting of polar glaciers; on the other hand, the problems of land pollution and ecological destruction caused by the accumulation of industrial by-products such as fly ash are increasingly prominent. Against this background, the development of carbon resource technology and industrial solid waste collaborative transformation systems has become a key issue for sustainable development [19,20]. By converting waste, such as carbon dioxide and fly ash in the atmosphere, into high value-added fuels, chemical raw materials, and new materials, it not only helps to alleviate the climate crisis and environmental pollution but also opens up an innovative path towards a clean energy supply. The carbon conversion technology matrix now functions as an integrated closed-loop system, combining direct air carbon capture [21], advanced carbon storage technology [22], and artificial photosynthesis systems mimicking natural processes [23]. This synergistic integration achieves circularity through engineered material solutions, which are exemplified by waste slag glass–ceramics [24,25,26]. These materials demonstrate the closed-loop mechanism by permanently immobilizing captured CO_2_ in mineral matrices while upcycling industrial waste, thereby seamlessly connecting carbon capture, geological storage, and photocatalytic conversion into a self-sustaining cycle. Yang et al. (Contribution 5) found that the introduction of tricalcium phosphate Ca_3_(PO_4_)_2_ in CaO-Al_2_O_3_-SiO_2_-Fe_2_O_3_-based high-temperature phase reconstruction composites could regulate the microstructure and performance of the glaze layer [14]. Through a 30 min sintering experiment at 1180 °C, it is shown that when the content of Ca_3_(PO_4_)_2_ is 8 wt%, the sample shows the best performance, with a water absorption rate as low as 0.03%; the Vickers microhardness reaches 6.5 GPa, indicating a high waterproof ability and wear resistance. The addition of Ca_3_(PO_4_)_2_ enhances the thermal expansion of the glaze layer and the blank by reducing the viscosity of the glaze layer, promoting the formation of the glass phase and regulating the precipitation of the crystal phase (such as quartz and white phosphate calcite), reducing the crack defects. In addition, the liquid–liquid phase separation structure induced by Ca_3_(PO_4_)_2_ and the microcrystalline phase synergistically produce a structural color effect through Rayley scattering (nanometer-scale phase separation) and Michel scattering (micron-level grain) to achieve a controlled adjustment of glaze opacity. This research provides theoretical and technical support for the surface modification and high-value application of solid waste-based ceramics. Liu et al. (Contribution 6) report the synthesis, characterization, and catalytic properties of a new binuclear gadolidenium (III) complex [Gd_2_(L)_6_(Phen)_2_]·4H_2_O (L = 4-acetylphenoxyacetic acid; Phen = 1,10-phyllorine) [27]. The complex is prepared via a water–ethanol solution reaction, and its structure is characterized by infrared spectroscopy (IR), ultraviolet–visible spectroscopy (UV-vis), thermogravimetric differential thermal analysis (TG-DSC), fluorescence spectroscopy, and single-crystal X-ray diffraction (SC-XRD). The results show that the complex is a trioblique crystal system (P-1 space group). Each Gd^3+^ ion forms a nine-coordination structure with the nitrogen atoms of two Phen ligands and the oxygen atoms of six L ligands. The one-dimensional chain and three-dimensional network structure is formed between molecules through benzene ring π-π stacking. The density functional theory (DFT) calculations show that the HOMO electron density is distributed in the L ligand, and the LUMO is located in the Phen ligand, indicating that there is a synergy between the ligands. Hirschfeld surface analysis shows that hydrogen–hydrogen (39.4%), oxygen–hydrogen (29.9%) and carbon–hydrogen (24.9%) interactions between molecules are the main ones, and the π-π stacking contribution accounts for only 4.2%. The fluorescence spectrum shows that the complex has characteristic emission peaks at 596 nm (^5^D_0_ → ^7^F_1_) and 620 nm (^5^D_0_ → ^7^F_2_), confirming that the energy from the ligand to Gd^3+^ demonstrates an efficient transfer of ions. In the catalytic performance test, the complex showed excellent photocatalytic CO_2_ reduction activity, with a CO yield of 41.5 μmol/g in 4 h, and the selectivity was >99%; in the oxidation reaction of benzene alcohol, with THF as the solvent, under the conditions of 120 °C and 0.5 MPa O_2_, the yield rate of benzaldehyde is 45.7%. After three cycles, the activity of the catalyst did not decrease significantly, showing good stability. The research provides a theoretical and experimental basis for the design of high-efficiency rare earth complex catalysts. In the future, the catalytic performance can be optimized by regulating ligands and metal centers. Zuo et al. (Contribution 7) significantly reviewed the enhanced performance of CO_2_ reduction in photocatalysts, which was achieved through defect regulation strategies [28]. For example, metal blanks (such as Zn blanks in ZnS) could reduce the reaction energy barrier and enhance CO_2_ adsorption ability, making HCOH selectivity as high as 86.6%; non-metallic defects (such as carbon gaps in g-C_3_N_4_) could optimize the energy band structure and promote the separation of photocarriers, and their CO yield reaches 4.18 mmol·g^−1^·h^−1^. In addition, composite defects (such as Fe-N co-doped TiO_2_) broadened the light absorption range through synergy, increasing the yield of CH_4_ by more than 10 times. Optimizing defect distribution (such as uniformly distributed oxygen vacancies) could maintain the stability of the catalyst and reduce the aggregation of active sites. Research showed that the precise regulation of defect type, concentration, and distribution can effectively enhance light absorption, charge separation efficiency, and CO_2_ adsorption activation ability, providing theoretical support and a technical path for the design of efficient and stable photocatalysts. The aforementioned research drives the realization of a closed-loop system integrating carbon capture–conversion–solid waste regeneration through multi-dimensional innovations in material modification (solid waste ceramics), molecular design (rare earth complexes), and defect regulation (photocatalysts). All these results provided key technological support for mitigating the climate crisis, supplying clean energy, and achieving the high-value utilization of industrial solid waste. Future efforts could focus on optimizing ligand/metal centers and implementing precise defect regulation strategies to further enhance system efficiency and scalability potential.

**Fuel Cell.** Against the backdrop of increasing energy demands and severe environmental challenges, direct ethanol fuel cells (DEFCs) have attracted significant attention due to their high energy conversion efficiency and the convenience of ethanol storage and transportation [29]. However, the large-scale commercial application of platinum-based catalysts in the ethanol oxidation reaction (EOR) has been limited by their high cost and susceptibility to CO poisoning via surface adsorption [30]. To address this issue, Su et al. (Contribution 8) successfully synthesized sub-nanoscale palladium-nickel@platinum-nickel (PdNi@PtNi) core–shell-structured nanoparticles [31]. This architecture not only enhanced atomic utilization efficiency but also optimized the d-band center and adsorbate binding energy through synergistic effects, thereby significantly improving the electrocatalytic performance and stability of the EOR. Experimental results demonstrated that PdNi@PtNi nanoparticles could exhibit exceptional catalytic activity and stability under alkaline conditions, offering novel insights for enhancing the electrocatalytic performance of platinum-based catalysts.

**Nitrogen Reduction Reaction (NRR).** Industrial ammonia synthesis relies on the Haber–Bosch process [32], which converts N_2_ and H_2_ into NH_3_ under high temperature and pressure conditions [33]. However, this method faces challenges such as high energy consumption and CO_2_ emissions [34,35] due to its dependence on fossil fuel-derived hydrogen production. Traditional ruthenium-based catalysts, although reducing activation energy, are limited by low efficiency in the NRR and competition from the HER, compromising selectivity and economic viability. Recent research has focused on ternary transition metal clusters (e.g., Co_3_-C_2_N), whose unique electronic structures optimize nitrogen adsorption pathways, significantly lowering the free energy of NRR while suppressing HER [36]. This enables efficient ammonia synthesis under ambient conditions. Such advancements promised substantial reductions in energy use and carbon emissions, driving the development of sustainable chemical processes. Xiao et al. (Contribution 9) investigated the electrocatalytic NRR performance of ternary transition metal clusters (M_3_-C_2_N) anchored on a C_2_N monolayer using density functional theory (DFT) calculations [37]. The research reveals that four catalysts, namely Co_3_-C_2_N, Cr_3_-C_2_N, Fe_3_-C_2_N, and Ni_3_-C_2_N, exhibited exceptional activity and selectivity in the NRR, with free energies lower than those of the Ru(0001) catalyst. These catalysts not only enabled the stable adsorption and activation of N_2_ molecules but also showed the ability to suppress the competing HER. These findings provided novel strategies for designing highly efficient and selective NRR catalysts, offering the potential for efficient ammonia synthesis under mild conditions.

**Supercapacitor.** Thanks to technological breakthroughs, such as the leading power density index, a service life far exceeding that of traditional batteries, and millisecond-level response speed, supercapacitors have become an important breakthrough in the upgrading of energy storage systems [38,39]. Fu et al. (Contribution 10) employed vacancy engineering to optimize selenium vacancies in NiCo_2_Se_4_ (Sev-NCS) [40]. The engineered samples exhibited enhanced electrochemical properties when applied as a supercapacitor electrode. The research team used ethylene glycol as a reducing agent in the NaOH alkaline environment, successfully prepared selenium-vacancy NiCo_2_Se_4_, and conducted in-depth research on its potential as a supercapacitor electrode material. Theoretical and experimental results showed that the introduction of selenium vacancies significantly changes the morphology and electronic structure of NiCo_2_ Se_4_, thus improving the conductivity of the material and the diffusion ability of electrolyte ions. The optimized Sev-NCS electrode showed a high specific capacitance of 2962.7 F·g^−1^ at a current density of 1 A·g^−1^, and still maintained a capacitance retention rate of 89.5% after 10,000 cycles, showing excellent cycle stability. An asymmetric device composed of an optimized Sev-NCS electrode as the positive electrode and activated carbon as the negative electrode achieved an energy density of 55.6 Wh·kg^−1^ at a power density of 800 W·kg^−1^. Therefore, this study provided new insights into the application of transition metal compound-based electrode materials in supercapacitors, demonstrating the great potential of vacancy engineering in improving electrochemical properties.

We sincerely hope that the studies contained in this Special Issue can stimulate more interdisciplinary cooperation and innovative research, as well as promoting the in-depth exploration of nanomaterials in relation to clean energy and sustainable development. Future research can pay more attention to the large-scale preparation, long-term stability, and multi-technology integration of materials to accelerate the transformation of laboratory results into industrial applications. It is expected that these cutting-edge explorations will inject new momentum into the realization of the global carbon neutrality goal and help build a green and low-carbon future energy system.
